# RETRACTED: Kulanayake et al. Regions of Bovine Adenovirus-3 Protein VII Involved in Interactions with Viral and Cellular Proteins. *Viruses* 2024, *16*, 732

**DOI:** 10.3390/v17121605

**Published:** 2025-12-12

**Authors:** Shermila Kulanayake, Faryal Dar, Suresh K. Tikoo

**Affiliations:** 1 Vaccinology & Immunotherapeutics Program, School of Public Health, University of Saskatchewan, Saskatoon, SK S7N 5E5, Canada; shermila.kulanayake@usask.ca (S.K.); uyl039@mail.usask.ca (F.D.); 2 Vaccine and Infectious Disease Organization (VIDO), University of Saskatchewan, Saskatoon, SK S7N 5E5, Canada; 3 Veterinary Microbiology, Western College of Veterinary Medicine, University of Saskatchewan, Saskatoon, SK S7N 5B4, Canada

The journal retracts the article, “Regions of Bovine Adenovirus-3 Protein VII Involved in Interactions with Viral and Cellular Proteins” [1], cited above.

Following publication, concerns were brought to the attention of the editorial office regarding multiple irregularities found in images presented in this article [1].

Adhering to our standard procedure, an investigation was conducted by the Editorial Office and Editorial Board, which confirmed indications of inappropriate irregularities in Figure 8B and partial overlap in sub-images included in Figures 7, 10B and 11B published in [1]. The authors collaborated during the investigation and provided supporting materials, however they were unable to satisfactorily explain mentioned irregularities. As a result, the Editorial Board has lost confidence in the reliability of the findings and decided to retract this publication [1], as per MDPI’s retraction policy (https://www.mdpi.com/ethics#_bookmark30).

This retraction was approved by the Editor-in-Chief of *Viruses* journal.

The authors did not agree with this retraction.

## References

[1] Kulanayake S., Dar F., Tikoo S.K. (2024). RETRACTED: Regions of Bovine Adenovirus-3 Protein VII Involved in Interactions with Viral and Cellular Proteins. Viruses.

